# Phycobiliproteins—A Family of Algae-Derived Biliproteins: Productions, Characterization and Pharmaceutical Potentials

**DOI:** 10.3390/md20070450

**Published:** 2022-07-09

**Authors:** Huaxin Chen, Hongtao Qi, Peng Xiong

**Affiliations:** 1 School of Life Sciences and Medicine, Shandong University of Technology, Zibo 255000, China; xiongp@sdut.edu.cn; 2 School of Life Sciences, Qingdao University, Qingdao 266000, China; qihongtao@qdu.edu.cn

**Keywords:** phycobiliprotein, phycobilin, biosynthesis, algae, pharmaceutical potentials

## Abstract

Phycobiliproteins (PBPs) are colored and water-soluble biliproteins found in cyanobacteria, rhodophytes, cryptomonads and cyanelles. They are divided into three main types: allophycocyanin, phycocyanin and phycoerythrin, according to their spectral properties. There are two methods for PBPs preparation. One is the extraction and purification of native PBPs from Cyanobacteria, Cryptophyta and Rhodophyta, and the other way is the production of recombinant PBPs by heterologous hosts. Apart from their function as light-harvesting antenna in photosynthesis, PBPs can be used as food colorants, nutraceuticals and fluorescent probes in immunofluorescence analysis. An increasing number of reports have revealed their pharmaceutical potentials such as antioxidant, anti-tumor, anti-inflammatory and antidiabetic effects. The advances in PBP biogenesis make it feasible to construct novel PBPs with various activities and produce recombinant PBPs by heterologous hosts at low cost. In this review, we present a critical overview on the productions, characterization and pharmaceutical potentials of PBPs, and discuss the key issues and future perspectives on the exploration of these valuable proteins.

## 1. Introduction

Natural compounds derived from algae exhibit a wide variety of biological activities. Many algae have been used as food or food additives for many years. In east Asian countries where algae have been utilized in cuisine and medicine, a lower incidence of chronic diseases, such as hyperlipidemia, coronary heart disease, diabetes and cancer, is observed compared to Western countries [1]. Algae are rich in sugars/fiber, proteins/peptides, lipids/fatty acids, minerals and vitamins. They are also abundant sources of secondary metabolites such as polysaccharides, sterols, tocopherols, terpenes, polyphenols, phycobilins and phycobiliproteins (PBPs). These compounds have been shown to possess antioxidant, anticancer, anti-inflammatory, antihypertensive, anti-hyperlipidemia, immunomodulatory, neuroprotective, antiviral and antimicrobial activities [2,3,4].

Among these bioactive compounds, PBPs have received much attention in the past few decades. PBPs are a family of colored and water-soluble biliproteins found in cyanobacteria, rhodophytes, cryptomonads and cyanelles [5]. They function as major light-harvesting antennas for absorption of light energy and transfer it into reaction centers of photosystems. Algae contribute to over 90% of the primary production in the oceans and play critical roles in the oceanic food chains and global carbon cycle. Their survival and flourishment in different habitats depend largely on solar radiation. Photosynthetically active radiation ranging from 400 nm to 700 nm is captured by PBPs and converted into chemical energy to support cell metabolisms. For efficient light absorption, several types of PBPs with distinct absorption spectra have evolved. PBPs have been developed as fluorescent probes in immunofluorescence assay due to their high extinction coefficient and fluorescence yield [6]. They are also used as natural colorants and food additives in chewing gum, ice sherbets, popsicles, candies, soft drinks, dairy products and cosmetics such as lipstick and eyeliner [7]. Furthermore, a vast number of investigations have revealed that PBPs possess various pharmaceutical potentials. The prices of PBPs from biotechnological companies are USD 5000 to USD 33,000/g [7]. Nowadays, drug development industries put more interest on PBP exploration for therapeutic purposes. According to a report of Future Market Insights, the PBP market value was USD 112.3 million in 2018 and is estimated to double in 2028 [8].

In this review, we describe the progress on PBP research including their structures, biosynthesis, production and bioactivities, with an emphasis on their pharmaceutical potentials. Based on the present state of research, we discuss the key issues and prospective for the further exploration of these PBPs.

## 2. Phycobiliproteins

**Figure 1:**
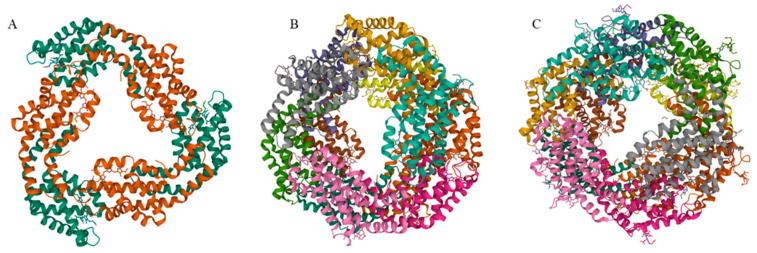
Ribbon model of the structure of PBPs. (**A**): 2V8A, the structure of *Thermosynechococcus elongatus* allophycocyanin at 3.5 Å, (**B**): 1HA7, The structure of *Spirulina Platensis* C-phycocyanin at 2.2 Å resolution., (**C**): 5NB3, High resolution C-phycoerythrin from marine cyanobacterium *Phormidium* sp. A09DM.

The basic building block of PBSs is monomers, which are heterodimers of two subunits (α and β). The β subunits are slightly larger than the α subunits. The association of three (αβ) monomers forms (αβ)_3_ trimers with a shape of an even circular disk. Except for APC, these trimers are quite stable and do not disassociate in low-ionic-strength solutions. Association of two trimers leads to formation of the (αβ)_6_ hexamers (Figure 1). For most types of PBPs, linker peptides participate in the trimer or hexamer assembly, contributing to the stability of the complexes.

Each subunit of PBPs carries one to three linear tetrapyrrole chromophores (phycobilins) at specific cysteine residues. The absorption spectra of PBPs are largely determined by the types of phycobilins, which include phycocyanobilin (PCB, λmax = 640 nm), phycoerythrobilin (PEB, λmax = 550 nm), phycourobilin (PUB, λmax = 490 nm), and phycoviolobilin (PVB, λmax = 590 nm). Based on their spectral properties, PBPs can be classified into three main types: allophycocyanin (APC), phycocyanin (PC), and phycoerythrin (PE). PBPs can be further classified into subtypes to distinguish their spectral properties. For example, PE is divided into R-PE, B-PE and C-PE; PC is divided into R-PC and C-PC. The prefixes to PBPs historically indicated their taxonomic source: C-, Cyanobacterial; B-, Bangiophycean; and R-, Rhodophytan, and later were used to denote spectral properties of PBPs [5].

### 2.1. Allophycocyanin

APC is located in the core of the phycobilisome, found in all phycobiliprotein-containing organisms. There are three subtypes of APC: allophycocyanin (APC), allophycocyanin-B (APC-B), and allophycocyanin core-membrane linker (APC-Lcm). Among the three subtypes, APC is the dominant PBP in the core of phycobilisome. APC assembles into core cylinders, while APC-B and APC-Lcm participate in the formation of two basal cylinders [9]. The α and β subunits of APC are highly conserved among different species and to a lesser extent between themselves [10]. Each α and β subunit bind a chromophore PCB through conserved cysteine residues α-84 and β-84. APC is typically isolated and purified as a trimer (αβ)_3_ and has an absorption maximum at 650 nm with a shoulder at 620 nm. However, when diluted to low concentrations, the trimeric APC dissociates to the αβ monomers. As a result, the maximal absorbance for the APC shifts from 650 nm to 618 nm. Correspondingly, the maximal fluorescence emission shifts from 660 nm to 643 nm [6].

In some cyanobacteria, the photosynthetic apparatuses can be remodeled in response to far-red light (FRL), facilitating efficient capturing of FRL for photosynthesis [11,12,13]. Recently, Li et al. presented a new phycobilisome-derived complex that consists only of allophycocyanin core subunits with red-shifted absorption peaks of 653 and 712 nm [14]. These red-shifted phycobiliprotein complexes were isolated from the chlorophyll f-containing cyanobacterium *Halomicronema hongdechloris*. It was demonstrated that the protein environment surrounding the pyrrole ring A of PCB on the APC alpha subunit is mostly responsible for the FRL absorbance. In addition, it was found that interactions between PCBs bound to alpha and beta subunits of adjacent protomers in trimeric APC complexes are responsible for a large bathochromic shift of about ~20 nm and notable sharpening of the long-wavelength absorbance band [15].

### 2.2. Phycocyanin

PCs are found in almost all phycobiliprotein-containing organisms, including cyanobacteria, red algae, glaucophytes, and some cryptophytes. Based on their spectral properties, PC is divided into three subtypes: (1) C-PC (λ max ~615–620 nm), found exclusively in Cyanobacteria, (2) phycoerythrocyanin (PEC, λ max ~575 nm), only found in some Cyanobacteria, and (3) R-PC (R-PC, λ max ~615 nm), mainly found in red algae [9]. PCs absorb light ranging from 580 nm to 630 nm and emit fluorescence with a maximum around 635–645 nm. In intact cells, PCs commonly exist in disk-shaped hexamers, and a rod-linker polypeptide or a rod-core-linker polypeptide is attached to the center cavity of hexamers. In general, a C-PC molecule carries a PCB at α-84 and two PCBs at β-84 and β-155. Analysis of energy transfer between chromophores has demonstrated that α-84 PCB and β-155 PCB in C-PC act as the excitation energy transfer donors and the β-84 PCB as the terminal acceptors. However, in some algal species, one or two peripheral chromophores (α-84 PCB and/or β-155 PCB) in PC are replaced by PVB, PEB, or PUB for adaptation to environmental conditions, which are rich in blue and green light [9,16,17]. When isolated from phycobilisome, PC exists as a hexameric structure (αβ)_6_ at pH 5.0–6.0 and a trimeric structure (αβ)3 at pH 7.0 [18]. C-PC is among the most studied PBPs because of its various biological and pharmacological properties [4]. 

### 2.3. Phycoerythrin

PEs are the most abundant PBPs in much red algae and in some unicellular cyanobacteria [6]. Compared with APC and PC, PE carries more phycobilins and therefore absorbs more light on an equal molar basis. The phycobilins attached to apo-PBPs can be PEB and PUB. Phycobilin contents differ for cyanobacteria living in either freshwater and soil or marine environments. PEs from freshwater and soil Cyanobacteria typically carry only PEB chromophores and exhibit absorbance spectra, with maxima around 565 nm [5]. Pes from marine unicellular *Synechococcus* sp. And *Synechocystis* sp. Strains bind the PUB chromophores at specific cysteine residues. Pes strongly absorb light, ranging from 480 nm to 570 nm, and emitted fluorescence from 575 to 580 nm. Based on the types of phycobilins and their spectral properties, PE is classified into three main subtypes: (1) B-phycoerythrin (B-PE) (λmax ~540–560 nm, shoulder ~495 nm), (2) R-phycoerythrin (R-PE) (λmax ~565, 545 and 495 nm), and (3) C-phycoerythrin (C-PE) (λmax ~563, 543 and ~492 nm). C-PEs are the most abundant PEs in cyanobacteria and can be further classified into two subtypes: C-PE-I and C-PE-II [5]. Typically, PE carried five chromophores with extra phycobilins linked to α-143 and a PEB doubly linked to β-50 and β-61 [19]. PE α and β subunits form into trimers and form disk-shaped hexamers through face-to-face aggregation with the help of a special linker peptide (named γ subunit). In some cyanobacterial species, the chromophore composition of PE can be changed in response to the light quality [20]. This process is known as complimentary chromatic adaptation and is proposed to be an important contributor to global primary productivity [21]. PEs exhibit quantum yields up to 0.98 and molar extinction coefficients of up to 2.4 × 10^6^. These exceptional spectral properties make PE a superior fluorescent probe over fluorescein and rhodamine, the most commonly used fluorescent dyes.

### 2.4. Biosynthesis of Phycobiliproteins

**Figure 2:**
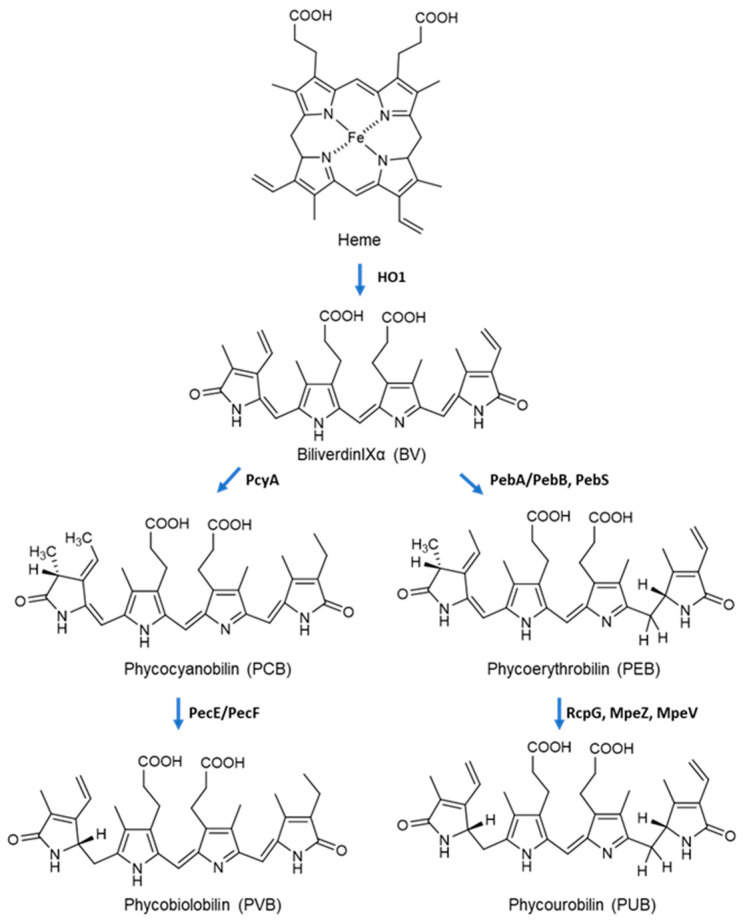
Biosynthesis of phycobilins in Cyanobacteria.

Biosynthesis of PBPs involves two processes: (1) expression of apo-PBPs and biosynthesis of phycobilin; (2) covalent attachment of phycobilin to apo-PBPs, which is mediated by various types of lyases. The phycobilins are derived from the heme metabolism (Figure 2). Under the action of heme oxygenase (HO1), heme is cleaved at the α-methene bridge and converted to biliverdin IXα (BV). One or more double bonds of the outer rings of BV can be reduced by ferredoxin-dependent bilin reductases. The phycocyanobilin: ferredoxin oxidoreductase (PcyA) catalyzes the transfer of four electrons from ferredoxin to the double bonds of BV to form PCB. 15,16-dihydrobiliverdin: ferredoxin oxidoreductase (PebA) transfers two electrons to biliverdin IXα to form 15,16-dihydrobiliverdin, which can be then converted to PEB via another two-electron reduction catalyzed by phycoerythrobilin:ferredoxin oxidoreductase (PebB). The formation of PVB and PUB is mediated by specific lyase-isomerases using bound-PCB and PEB as substrate, respectively [22,23]. 

The attachment of phycobilin to apo-PBPs is mediated by autocatalytic or lyase-catalyzed binding. ApcE has lyase domains in its N-terminal region and can attach PCB autocatalytically to form native-like LCM [24]. For lyases, CpcE/CpcF is the first identified enzyme that catalyzes the attachment of PCB to α-84 of apo-CpcA [25]. It also catalyzes the addition of PEB to apo-CpcA, but with reduced affinity and kinetics compared with PCB. A number of lyases, including CpcS/CpcU [26], CpcT [27], MpeZ [23], MpeU [28], CpeF [29], MpeV [30], have been identified in recent years. However, there are many lyases yet to be discovered due to complexity of the large PBP family.

## 3. Production of Phycobiliproteins

Many efforts have been made on the efficient production of PBPs. There are two methods for PBP preparation. The conventional way is the extraction and purification of native PBPs from Cyanobacteria, Cryptophyta and Rhodophyta [4]. The bioactivities of PBPs reported in the literature are based mainly on native PBPs. With the progress in recombinant DNA technology, the complete pathways for PBPs have been successfully constructed in engineered heterologous hosts. A number of PBPs have been produced in *E. coli* (Table 1). Compared with native PBPs, a single subunit of α or β can be prepared more easily from engineered hosts and may offer some advantages in therapeutic use due to its small size. However, at present, less work is focused on the evaluation of recombinant PBPs and much attention should be paid to the recombinant PBPs in the future.

### 3.1. Native Phycobiliproteins

The main producers for PBP are cyanobacterium *Arthrospira platensis* (formerly *Spirulina platensis*) and the red microalga *Porphyridium* (Rhodophyta) [31]. Other algal species such as *Synechococcus* sp., *Limnothrix* sp. (Cyanobacteria) and *Neopyropia yezoensis* (formerly *Porphyra yezoensis*) (Rhodophyta) are also the sources for PBP preparations. *A*. *Platensis* can grow rapidly in open ponds in alkaline conditions, under which conditions most algae hardly grow. In 2021, a production of 10,000 tons of dry *A. platensis* cell mass is estimated in China. Environmental factors, particularly light, nitrogen and carbon sources, affect algal growth and PBP productions. In large-scale cultivations of cyanobacteria, poor permeation of light into deeper layers of water is the major problem that hampers high cell density cultivations. In open ponds, light is in excess of what is required for photosynthesis for algal cell growth in the surface layer, while in the deep layers, algal growth is limited by low light. The problem is partially solved by using well-designed photobioreactors, which provide equal light distribution in the cultures. Low and medium light is preferred to obtain higher productions of PBPs. Nitrogen is another important element for the production of PBPs, which act as the main storage of nitrogen algal cells under stress conditions. The common nitrogen sources for algae cultivation are nitrate, ammonium and urea. However, the preferred nitrogen sources may vary from species to species. It has been shown that some species such as *Phormidium* sp. and *Pseudoscillatoria* sp. (Cyanobacteria) preferred ammonium, while *A. platensis* grow better when nitrate is added into the media [32]. Carbon source along with other factors including temperature, pH, and salinity also influence the production of PBPs. Therefore, for a given algal species, it is necessary to optimize these factors to maximize PBP production.

Methods for native PBP purification depend on algal species and the types of PBPs. Since PBPs strongly absorb visible light, their purity can be determined by the ratio of maximum absorbance to the absorbance at 280 nm. For C-PC, it is considered to be food grade, cosmetic grade, reactive grade and analytical grade when this ratio is >0.7, 1.5, 3.9 and 4.0, respectively [4]. In general, PBP purification involves two steps. The initial step is the extraction of PBP from algal cells. Different methods are adopted in practice to disrupt algal cells, including chemical treatment, physical treatment (freezing and thawing, grinding, high-pressure homogenization, ultrasonication, etc.) and enzymatic treatment (lysozyme digestion). Novel extraction techniques such as ultrasound-assisted extraction, microwave-assisted extraction, high pressure processing, pulsed electric fields and supercritical fluid extraction have been developed in recent years [33]. A proper method should ensure high efficiency of cell disruption and intact structure of PBPs with relatively low cost. Among these methods, freezing–thawing has been shown to be effective in disruption of cyanobacteria [34,35].

The second step is the purification of PBPs from crude extractions by multiple separation processes including ammonium sulfate precipitation, chromatography, membrane filtration or two-phase aqueous extraction. Ammonium sulfate precipitation is a simple process conducted in the initial purification stage to concentrate the PBP samples and remove most undesirable components. Generally, 20–30% of ammonium sulfate precipitates the undesirable proteins, which can be removed by centrifugation. The supernatant is treated with 60–70% ammonium sulfate, leading to precipitation of PBPs. To further improve the purity, chromatographic separation is required. Ion exchange chromatography has been shown to be effective for PBP purification [36,37]. Elution of PBPs works through a gradient elution of ionic strength, or a gradient of pH is efficient for PBP separation [38]. Other chromatographic techniques such as hydroxyapatite chromatography, hydrophobic chromatography and gel filtration chromatography are also adopted in PBP separation. Membrane filtration can concentrate PBP crude extract and increase the purity of PBPs [39]. A critical factor is the selection of a proper membrane, of which the cut-off value is suitable to reach the targeted degree of purity index [4]. Aqueous two-phase extraction is easy to scale up but less efficient compared to the conventional purification processes. This technique may be suitable to prepare a large amount of PBPs at low cost [40].

**Table 1:** Production of recombinant apo- or holo-phycobiliproteins.

PBPs	Sources for PBP Gene	Hosts	Phycobilins	Lyases	Chromophorylation (%)	Reference
Apo-ApcA, Apo-ApcB	*Synechococcos* sp. PCC 7002	*E. coli*	PCB	-	-	[41]
Apo-APC	*Asterocapsa nidulans* (formerly *Anacystis nidulans*)	*E. coli*	PCB	-	-	[42]
Apo-CpcA	*Anacystis nidufuns* R2	*E. coli*	PCB	-	-	[43]
Holo-CpcA	*Synechocystis* sp. PCC6803	*E. col*	PCB	CpcE/CpcF	~33.3%	[44]
Holo-PecA	*Anabaena* sp. PCC7120	*E. coli*	PVB	PecE/PecF	n.d.	[45]
Holo-ApcAB	*Synechococcus* sp. PCC 7002	*E. coli*	PCB	CpcU/CpcS	71.9%	[46]
Holo-ApcAB	*Synechocystis* sp. PCC 6803	*E. coli*	PCB	CpcU/CpcS	n.d.	[47]
Holo-ApcAB	*Gracilaria chilensis* (formerly *Agarophyton chilense*)	*E. coli*	PCB	CpcU/CpcS	52% and 57%	[48]
Holo-ApcA	*Synechococcus elongatus* BP-1	*E. coli*	PCB	CpcS	n.d.	[46]
Holo-ApcB	*Spirulina* sp.	*E. coli*	PCB	CpcS	81.4%	[49]
Holo-ApcA	*Synechococcus elongatus* BP-1	*E. coli*	PCB	CpcS	n.d.	[50]
Streptavidin-Holo-ApcA	*Synechococcus elongatus* BP-1	*E. coli*	PCB or PEB	CpcS	n.d.	[51]
Streptavidin-Holo-ApcA	*Synechococcus elongatus* BP-1	*E. coli*	PEB	CpcS	Up to 98.6%	[52]
Holo-ApcB	*Synechococcus elongatus* BP-1	*E. coli*	PCB	CpcS	n.d.	[53]
Holo-ApcF	*Synechococcus* sp. PCC 7002	*E. coli*	PCB	CpcU/CpcS	68.1%	[46]
Holo-CpcA	*Synechocystis* sp. PCC 6803	*E. coli*	PCB	CpcE/CpcF	48.1%	[46]
Holo-CpcB	*Synechocystis* sp. PCC 6803	*E. coli*	PCB	CpcU/CpcS	37.1	[46]
Holo-CpcB	*Synechocystis* sp. PCC 6803	*E. coli*	PCB	CpcT	17.4	[46]
Holo-CpcB	*Synechococcuselongatus* BP-1	*E. coli*	PEB, PUB	CpcU, CpcT	n.d.	[54]
Holo-CpcA	*Synechocystis* sp. PCC 6803 *Synechococcus* sp. PCC 7002	*E. coli*	PCB, PEB, PΦB, PUB, PVB, PtVB	CpcE/CpcF PecE/PecF	n.d.	[55]
Holo-CpeA	*Microchaete diplosiphon* (formerly *Fremyella diplosiphon*)	*E. coli*	PEB	CpeY	n.d.	[56]
Holo-CpeB	*Synechococcus* sp. RS9916	*E. coli*	PUB	MpeV	n.d.	[30]
Holo-CpeB	*Microchaete diplosiphon* (formerly *Fremyella diplosiphon*)	*E. coli*	PEB	CpeF	n.d	[29]
Holo-CpeB	*Prochlorococcus marinus* MED4 (Cyanobacteria)	*E. coli*	PEB	CpeS	n.d.	[57]
PcA/PcB	*Gracilariopsis lemaneiformis* (Rhodophyta)	*E. coli*	PCB	CpcU/CpcS, CpcE/CpcF, CpcT	n.d.	[58]
Holo-MpeA	*Synechococcus* sp. RS9916	*E. coli*	PUB	MpeZ	n.d.	[23]
Holo-C-PC equipped with different tags	*Anabaena* sp. PCC7120	*Anabaena* sp. PCC7120	PCB	-	n.d.	[59]
Holo-APC	*Cyanophora paradoxa* (Glaucophyta)	*Synechococcus* sp. PCC 7002	PCB	-	n.d.	[41]

### 3.2. Recombinant Phycobiliproteins

Biosynthesis of PBPs in a heterologous host offers an efficient method for the production of recombinant PBPs. As early as in the 1980s, genes coding for PC and APC were cloned, characterized and successfully expressed in *E. coli* or cyanobacteria. The expression of PC α and β subunit was probably driven by s native promoter centered about 374 bp upstream from the translation start for *cpcB*. The *apcA* and *apcB* genes of *Cyanophora paradoxa* (Glaucophyta) were expressed in *E. coli* under control of its native promoter. Lau et al. [43] reported that the *cpcA* gene from *Asterocapsa nidulans* (formerly *Anacystis nidulans*) was expressed in *E. coli* under the control of *lacZ* promoter of pUC8 vector. The expression level of the apo-CpcA was estimated to be between 0.5–1% of the total soluble proteins in *E. coli* cells. These early studies presented the successful expression of PBPs in *E. coli* and showed that recombinant PBPs were stable in this host. However, these PBPs were expressed in the apo-protein form. In a study by Lormimier et al. [41], the *apcA* and *apcB* gene from Cyanophora *paradoxa* (Glaucophyta) were transferred to the *Synechococcus* sp. PCC 7002 on plasmid replicon. The resulting showed that PBPs isolated from the transformed cells contained *C. paradoxa* APC subunits, which covalently carried a chromophore and were incorporated into the light-harvesting apparatus.

In a milestone work reported by Tooley et al. [44], the entire pathway for the biosynthesis of holo-CpcA from cyanobacterial *Synechocystis* sp. PCC6803 was reconstituted in *E. coli*. The cyanobacterial genes responsible for PCB biosynthesis from heme was expressed from a plasmid under control of the hybrid trp-lac promoter. The genes coding for lyases (CpcE and CpcF) and CpcA were co-expressed from a second plasmid harboring a tra promoter. The recombinant *E. coli* cells produced holo-CpcA with spectroscopic properties similar to those of the same protein isolated from cyanobacteria. In another work, Tooley et al. showed that phycoerythrocyanin holo-α subunit could be produced in *E. coli* in a similar way [45]. From then on, a number of holo-PBPs were produced in recombinant *E. coli* cells. Guan et al. [60] reconstituted the pathway of holo-CpcA in *E. coli* by using one expression vector. In this work, an expression vector containing five essential genes for holo-CpcA biosynthesis was constructed. In the expression vector, the genes *HO1* and *pcyA* were designed as an operon and then inserted into the second cassette of plasmid while *cpcA* together with *cpcE* and *cpcF* were designed as another operon and then inserted into the first cassette of the plasmid. The “one expression vector” strategy may offer better plasmid stability as compared to the “multiple expression vectors” strategy, which is important to maintain the full holo-CpcA pathway in *E. coli*. In addition, selection by only one antibiotic is cost-saving, especially for large-scale cultivations. These recombinant PBPs can be easily purified by using immobilized metal affinity chromatography, due to the addition of 6×His tag to N terminals of the recombinant PBPs.

In *E. coli*, the PBP lyases are less specific to phycobilins as they could catalyze attachment of noncognate phycobilins to apo-PBPs. Namely, PBPs carrying noncognate phycobilins can be produced in recombinant *E. coli* cells. In a work by Alvey et al. [55], *cpcA* from *Synechocystis* sp. PCC 6803 and *Synechococcus* sp. PCC 7002 was coexpressed with the *cpcE/cpcF* from *Synechocystis* sp. PCC 6803 or *pecE/pecF* from *Noctoc* sp. PCC 7120. Both lyases were capable of attaching three different phycobilins (PCB, PEB and PVB) to CpcA. Therefore, six different CpcA variants, each with a unique phycobilin, could be produced in *E. coli* cells. In our previous work [51], *cpcA* from thermophilic cyanobacterium *Thermosynechococcus vestitus* BP-1 (formerly *Thermosynechococcus elongatus* BP-1), together with the *cpcS*, *HO1* and *pebS* were co-expressed in *E. coli*. Holo-ApcA-carrying PEB was successfully produced, which showed a distinct spectral property from native holo-ApcA. PBPs are regarded as potential photosensitizers in cancer therapy [61]. The ability to produce unnatural PBP in *E. coli* would expand the types of PBPs and contribute to the exploration of novel photosensitizers.

The main shortage of recombinant holo-PBPs produced in *E. coli* is the inefficiency of chromophorylation. Tooley et al. [44] showed that about a third of the apo-CpcA was converted to holo-CpcA in *E. coli*. Ge et al. reported a fraction of 81.4% for HT-ApcB produced in *E. coli* [49]. *Biswas* et al. [46] presented a systemic study on the chromophorylation efficiency and specificity of all bilin lyases from *Synechococcus* sp. strain PCC 7002. The recombinant holo-proteins included HT-CpcA, HT-CpcB, HT-ApcA/ApcB, HT-ApcD, HT-ApcF and GST-ApcE. The percentage of chromophorylation for these holo-proteins ranged from 17.4% to 71.9%, indicating inefficient chromophorylation for the proteins reconstituted in *E. coli*. The addition of aminolaevulinic acid or iron did not improve production of PCB and holo-protein [45,62]; it is unlikely that chromophorylation is limited by heme availability. Instead, the incomplete chromophorylation might be due to unfavorable codon usage or due to the aggregation of the recombinant proteins into insoluble inclusion bodies [44]. In our previous work, the codon for *cpcS* was optimized for *E. coli*, leading to an increase in expression level of *cpcS* and improvement of chromophorylation of the recombinant PBP. In addition, we showed that plasmid stability is also an important factor limiting the efficient chromophorylation of recombinant PBPs [52]. Compared with multiple plasmids, a single plasmid full of the entire PBP pathway is preferred, which would contribute to the maintenance of the entire heterologous pathway during cell divisions and avoid too much antibiotic selection markers.

At present, recombinant PBPs are expressed mostly in *E. coli* (Table 1). Fewer studies are performed in eukaryotic hosts. Industrial microbes such as *Saccharomyces cerevisiae* and *Pichia pastoris* would be good candidates for the production of PBPs. The vast progress in genetic manipulation approaches such as CRISPR make the construction of a stable PBP pathway in these hosts more convenient and would promote the mass production of the valuable PBPs.

## 4. Pharmaceutical Potentials

**Figure 3:**
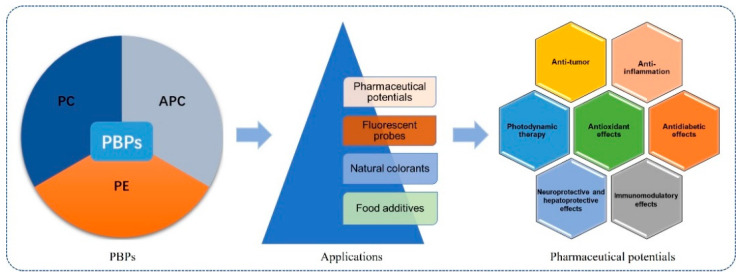
Application as food additives, natural colorants, fluorescent probes and the pharmaceutical potentials of PBPs (APC, PC and PE).

Native PBPs have been utilized as food additives, natural colorants and fluorescent probes for tens of years. Much work has been carried out to evaluate their pharmaceutical potentials. PBPs exhibit various bioactivities, such as antioxidant, anti-tumor, neuroprotective and hepatoprotective properties, and could be developed as photosensitizers in tumor therapy (Figure 3).

### 4.1. Antioxidant Effects

Oxidative stresses result from reactive oxygen species (ROS). In cases that a decline in antioxidant defense or increase in production of reactive species occurs, the accumulation of reactive species will cause damage to macromolecules, such as proteins, DNA and lipids, and thus lead to abnormal cellular metabolism and even cell death.

The antioxidant activity of PBP was firstly demonstrated by in vitro and in vivo assays by Romay et al. [63]. C-PC from *Arthospira maxima* was able to scavenge alkoxy radicals (RO•, IC_50_ = 76 mg/mL) and hydroxyl radicals (OH•, IC_50_ = 0:91 mg/mL). C-PC also inhibited luminol-enhanced chemiluminescence from zymosan-activated human polymorphonuclear leukocytes, microsomal lipid peroxidation (IC_50_ = 12 mg/mL) induced by Fe^3+^-ascorbic acid, and the glucose-oxidase-induced inflammation in mouse paw, a model of inflammatory response in which peroxide and hydroxyl radicals are involved. Further study showed that C-PC exhibited anti-inflammatory activity in four experimental models of inflammation. Such anti-inflammatory activity could be, at least partially, explained by the antioxidative and oxygen free radical scavenging properties [64]. C-PC from *A. platensis* effectively inhibited CCl_4_-induced lipid peroxidation in rat liver in vivo [65]. Both native and reduced PC significantly inhibited peroxyl radical-induced lipid peroxidation in rat liver microsomes and the inhibition was concentration-dependent with an IC_50_ of 11.35 and 12.7 mM, respectively. The results suggested that the covalently linked PCB is involved in the antioxidant and radical scavenging activity of PC. Sonani et al. [66] described the in vitro antioxidant activity of three major PBPs, which were isolated from the marine cyanobacterium Lyngby asp. A09DM. The results showed significant and dose-dependent antioxidant activities of all PBPs in an order of PE > PC > APC. The potential application of PE as antioxidant needs deep investigation in the future.

Direct evidence for the involvement of phycobilins in antioxidant activity was shown by PCB prepared from PBPs. When the concentrations of PC and PCB prepared from *A. platensis* were equal on a phycobilin basis, the antioxidant activity was almost the same as that of PC in the AAPH-containing reaction mixture, indicating that PCB accounted for the majority of the antioxidant activity [67]. PC and PCB also exhibited antioxidant activity against peroxynitrite (ONOO-). Scavenging of ONOO- by PC and PCB was established by examining their interactions. The relative antioxidant ratio and IC50 value indicated that PC is a more efficient ONOO- scavenger than PCB [68]. PC and PCB derived from Aphanizomenon flos-aquae was shown to have similar activities against peroxyl radicals, and higher activities than well-known antioxidants such as Trolox, ascorbic acid and reduced glutathione [69]. These findings indicate that PCB is mostly responsible for the antioxidant activity of PC. Recently, phycobilins including PCB, PEB, PUB and PVB was reported to potent phytochemical inhibitors to Mpro and PLpro proteases of SARS-CoV-2 [70].

The structure of phycobilin is similar to that of bilirubin, a physiological and chain-breaking antioxidant [71]. This similarity may explain the antioxidant activities of PBPs. However, a number of studies have demonstrated that apo-PBPs, which do not carry phycobilin chromophore, were able to quench ROS. Ge et al. [72] expressed α subunit (6×His-apo-ApcA), β subunit (6×His-apo-ApcB) and 6×His-apo-ApcAB from *Asterocapsa nidulans* (formerly *Anacystis nidulans*) UTEX 625 in *E. coli*. The results showed that recombinant apo-ApcA and apo-ApcB had stronger antioxidant activities than native APC and apo-ApcAB. It is proposed that the combination of α and β subunits covered the active domain in the subunit and thus reduced the antioxidant activities. In another study, recombinant apo-ApcA and holo-ApcA from *Synechocystis* sp. PCC6803 were produced in *E. coli* [73]. Like native APC, both proteins exhibited hydroxyl radical and peroxyl radical scavenging activities, with a descending order of recombinant holo-ApcA > recombinant apo-ApcA > native APC. However, Pleonsil et al. [74] showed that the antioxidant ability of apo-CpcB from *A. platensis* was much lower than that of native PC. Nevertheless, the antioxidant activities of apo-PBP indicate that some active sites beyond phycobilin are located on the PBP. The active sites may be related to sulfur-containing amino acids [75]. It has been shown that cysteine and methionine residues, especially when located on the protein surface, play important roles in protecting the cell from oxidative damage through its thiol functional group [76]. The apo-CpcA contains four cysteine and six methionine residues. These amino acid residues may contribute to the antioxidant activity of the apo-protein [74]. Alternatively, PBPs were believed to chelate and reduce the ferrous ion efficiently, implying the combined involvement of both electron-donating and metal-ion-chelating ability of the PBP-constituting amino acids in expressing antioxidant activity [67]. For example, an amino acid with a hydrophobic side chain is good proton donor and metal ion chelator. Similarly, acidic, basic and aromatic amino acids are supposed to sequester metal ions. It is hypothesized that the antioxidant action of PBPs differs depending on different mechanisms associated with side chains of the various constituting amino acids [67].

The antioxidant effects are affected by several factors such as light, pH and denaturing agents. When exposed to light, PC produced hydroxyl radicals while in the dark PC scavenged the hydroxyl radicals. An increase in pH above 7.0 or denature of PC by sodium dodecyl sulfate or urea led to the loss of the ability to produce hydroxyl radicals, and concurrently an increase in antioxidant capacity [77]. Moreover, the trypsin-digested fragments of apo-PC exhibited antioxidant activities. These data indicate that some active sites are buried in the native conformation and show antioxidant activities when exposed on the surface of apo-proteins.

### 4.2. Anti-Tumor Effects

Cancer is one of the main diseases causing death in the world. At the cellular level, cancer cells are characterized by indefinite cell proliferation, disability of apoptosis and invasion of cell growth. Therefore, cancer therapy can be achieved through inhibition of tumor cell proliferation, induction of tumor cell apoptosis and cell cycle arrest, and limitation of tumor cell migration. Increasing evidence has confirmed the inhibiting effects of PBP on different types of cancer, including breast cancer, liver cancer, lung cancer, colon cancer, leukemia and bone marrow cancer. The effective dosages of PBP on cancer might differ, depending on the tumor cell lines [78,79,80,81]. Notably, high dosages of PBP did not induce significant adverse effects or mortality in animal experiments [82,83].

Regulation of the cell cycle is critical for cell proliferation, differentiation and apoptosis. The development of cancer is closely associated with dysfunction of cell cycle regulation [84]. PBPs can affect the cell cycle, causing cell cycle arrest. Liu et al. [85] reported the inhibitory effect of C-PC from *A. platensis* on the growth of human chronic myelogenous leukemia-blast crisis K562 cells. These cells were stopped to progress through S-phase and arrested at the G1 phase. The treatment of HT-29 and A549 cells with C-PC from *Oscillatoria tenuis* led to a decrease in the G2/M phase compared with the control. The percentage of S phase in treated cells increased and concurrently the percentage of cells in G0/G1 phase increased. Flow cytometry analysis also revealed the effect of C-PC on the accumulation of cells in the G0/G1 phases. These findings indicated that C-PC led to cell cycle arrest in the G0/G1 phases in cancer cells, HT-29, and A549 cells. Jiang et al. [86] constructed tumor-targeted nano-drug C-PC/CMC-CD59sp nanoparticles. These nanoparticles are composed of carbocymethyl chitosan (CMC), C-PC and CD59-specific ligand peptide (CD59sp) and were found to induce G0/G1 cell cycle arrest and inhibit the proliferation in cervical cancer HeLa and SiHa cell. In vivo experimentation showed that the cell cycle was regulated via up-regulating p21 expression and down-regulating the expression of Cyclin D1 and CDK4 [86].

Induction of tumor cell apoptosis is an important strategy to treat cancer. Early research showed that C-PC treatment of human chronic myeloid leukemia cell line K562 led to the release of cytochrome c into the cytosol and poly ribose polymerase cleavage. The results also showed down-regulation of anti-apoptotic Bcl-2 but without any changes in pro-apoptotic Bax and thereby changing the Bcl-2/Bax ratio towards apoptosis [87]. Li et al. [88] reported that the growth of HeLa cells was inhibited by C-PC treatment in a dose-dependent manner. Electron-microscopic examination showed that C-PC could induce characteristic apoptotic features, including cell shrinkage, membrane blebbing, microvilli loss, chromatin margination and condensation into dense granules or blocks. Agarose electrophoresis of genomic DNA of C-PC-treated HeLa cells showed a fragmentation pattern typical for apoptotic cells. Flow-cytometric analysis of HeLa cells treated with different concentrations of C-PC demonstrated an increasing percentage of cells in the sub-G0/G1 phase. Moreover, Caspases 2, 3, 4, 6, 8, 9, and 10 were activated in C-PC-treated HeLa cells. These results indicated that C-PC down-regulated the anti-apoptotic gene, activated pro-apoptotic gene expression and then facilitated the transduction of apoptosis signals [88]. Recently, Jiang et al., [84] found that C-PC effectively inhibited MDA-MB-231 cell proliferation, induced cell apoptosis and triggered G0/G1 cell cycle arrest. C-PC- mediated apoptosis was regulated by the inhibition of the ERK pathway and the activation of the JNK pathway and p38 MAPK pathway.

Recent reports showed that PBP could inhibit epithelial-to-mesenchymal transition [89,90]. C-PC inhibited epithelial-to-mesenchymal transition (EMT) in human cervical cancer Caski cells by up-regulating E-cadherin expression and down-regulating N-cadherin expression. The expression of Twist, Snail and Zeb1 transcription factors related to EMT was also down-regulated. The data revealed that C-PC reversed TGF-β1-induced EMT in cervical cancer cells and down-regulated the TGF-β/samd signaling pathway-induced G0/G1 arrest of tumor cell cycle [90].

Interestingly, recombinant subunits of PBP, which were expressed in *E. coli* in their apo form, were also found to have anti-tumor activities. Treatment with recombinant apo-CpcB (rCpcB) inhibited four tumor cell lines’ proliferation and induced apoptosis and led to the depolymerization of microtubules and actin-filaments. rCpcB interacted with membrane-associated β-tubulin and glyceraldehyde-3-phosphate dehydrogenase (GAPDH). In the treated cells, caspase-3 ad caspase-8 activities increased and nuclear level of GAPGH decreased significantly. This study indicated that the molecular mechanism of rCpcB-mediated tumor cell inhibition may be different from that of the whole C-PC [91]. Recombinant apo-APC was also reported to be able to inhibit H22 hepatoma in mice, with inhibition rates ranging from 36% to 62% with dosages from 6.25 to 50 mg/kg/day [49].

PBPs can be used in combination with chemotherapy drugs to improve the safety and efficacy and reduce dosage of the single drug during cancer therapy [84]. Gantar et al. [92] revealed the synergic effects of C-PC from *Limnothrix* sp. 37-2-1 (Cyanobacteria) with topotecan (TPT) on Prostate Cell Line (LNCaP). When a low dosage of TPT was combined with C-PC, the cancer cells were killed at a higher rate than when TPT was used alone at full dose. The use of two compounds together increased the level of radical oxygen species and activated the activities of caspase-9 and caspase-3, induced apoptosis of tumor cells, and diminished side effects of topotecan [92]. Yang et al. [93] reported that combined use with ATRA and C-PC significantly reduced the dose and side effects of ATRA on HeLa cells. The combination therapy down-regulated anti-apoptotic protein Bcl-2, up-regulated the expression of pro-apoptotic Caspase-3 protein, inhibited Cyclin D1, cell-cycle-related CDK-4 and complement regulatory protein CD59 expression and induced the HeLa cell apoptosis. Bingula et al. showed that when lung cancer A549 cells were treated with a combination of betaine and C-PC, an up to 60% decrease in viability was observed, which is significant compared with betaine (50%) or C-PC treatment alone. Combined treatment reduced the stimulation of NF-κB expression by TNF-α, increased the amount of the proapoptotic p38 MAPK, and induced a cell cycle arrest in G2/M phase for ~60% of cells.

At present, PBPs are not used in clinical cancer treatment, possibly due to the efficacy not being high enough. In addition, the short in vivo half-life of PBPs limits their application as anti-cancer drugs. From the literatures described above and recent reviews [4,72,84], it is obvious that anti-tumor activities and their underlying mechanisms were mostly evaluated using C-PCs as the drugs. APC and PE are spectrally and somewhat structurally different from C-PC, which might offer novel activities against tumors. In this respect, it is of significance to examine the actions of APC and PE in tumor therapy in future work.

### 4.3. Anti-Inflammatory Effects

It was reported by Remirez et al. [94] that C-PC from *Arthospira maxima* exhibited an anti-inflammatory effect in azymosan-induced arthritis model in mice. C-PC reduced in a dose-dependent manner ear oedema induced by arachidonic acid and 12-O-tetradecanoyl phorbol-13-acetate in mice as well as carrageenan-induced rat paw oedema. These anti-inflammatory activities may be related to the antioxidative properties and down-regulation of cytokine secretion and arachidonic acid mechanism. Later studies showed that PBPs exhibited anti-inflammatory activities in various models such as glucose-oxidase-induced inflammation in mouse paw, in carrageenan-induced rat paw edema, arachidonic acid- and tetradecanoylphorbol acetate-induced ear edema model in mice, zymosan-induced experimental arthritis in mice, and acetic-acid-induced colitis in rats [95]. Isolated enzyme and whole-blood assays indicated that C-PC from *Spirulina platensis* is a selective inhibitor of cyclooxygenase-2 (COX-2), which is upregulated during inflammation. Reduced PC and PCB are poor inhibitors of COX-2 without selectivity, implying that apoprotein plays a key role in the selective inhibition of COX-2 [96]. In cyclophosphamide (CYP)-induced cystitis in mice, C-PC relieved symptoms by inhibiting bladder inflammation through COX-2 and EP4 expression [97].

### 4.4. Antidiabetic Effects

Diabetes mellitus is a metabolic disorder characterized by hyperglycemia and alterations in carbohydrate, fat and protein metabolism. In streptozotocine-induced type 2 diabetic rats, C-PE was found to ameliorate diabetic complications by reducing the oxidative stress and the oxidized low-density lipoprotein-triggered atherogenesis [98]. Administration of C-PE reduced food intake, organ weights, serum concentrations of glucose, and cholesterol, and increased body weight, total protein, bilirubin and ferric-reducing ability of plasma values. In addition, hepatic and renal tissues demonstrated significant decreases in TBARS, lipid hydroperoxide and conjugated diene contents, with increases in superoxide dismutase, catalase, glutathione peroxidase, reduced glutathione, vitamin E and vitamin C levels [98].

The administration of PC significantly decreased the body weight, fasting plasma glucose, and 24 h random blood glucose levels, and suppressed the abnormal enlargement of islets observed in the pancreas of KKAy mice. It was proposed that the antidiabetic effect of C-PC on KKAy mice is related to its ability to improve insulin sensitivity, reduce insulin resistance of peripheral target tissues and regulate glucolipide metabolism [99]. In db/db mice, a rodent model for type 2 diabetes, it was showed that oral administration of C-PC (300 mgkg^−1^ for 10 weeks) protected against albuminuria and renal mesangial expansion, and normalized urinary and renal oxidative stress markers and the expression of NAD(P)H oxidase components. Thus, it is concluded that oral administration of PC and PCB may offer a novel and feasible therapeutic approach against diabetic nephropathy [100].

Recent study suggested that the antidiabetic activity might be related to inhibition of α-amylase and α-glucosidase. An in silico analysis predicted the molecular interaction between PC and α-amylase and α-glucosidase enzymes. Molecular docking simulations indicated that PC inhibits the enzymes by binding to the active site and causing a disruption on substrate-enzyme binding. PC seems to play a crucial role in establishing the interaction within the cavity of active sites of the two enzymes [101].

### 4.5. Neuroprotective and Hepatoprotective Effects

The neuroprotective role of PC was demonstrated in kainate-injured brains of rats [102]. Oral administration of C-PC reduced microglial and astroglial activation induced by kainic acid, indicating that some metabolites of this protein crossed the hemato-encephalic barrier and exerted antioxidant effects in the hippocampus. It is suggested that C-PC could be used to treat oxidative stress-induced neuronal injury in neurodegenerative diseases, such as Alzheimer’s and Parkinson’s. In rat cerebellar granule cell cultures, PC showed a neuroprotective effect against 24 h of potassium and serum deprivation and prevented potassium/serum deprivation-induced apoptosis [103].

Pentón-Rol et al. [104] demonstrated that C-PC given either prophylactically or therapeutically was able to significantly reduce the infarct volume. In addition, C-PC exhibited a protective effect against hippocampus neuronal cell death and prevented the lipid peroxidation and increased ferric reducing ability of plasma in serum and brain homogenates. These findings suggest that C-PC may represent a potential preventive and acute disease-modifying pharmacological agent for stroke therapy. In SH-SY5Y neuronal cells, tert-butylhydroperoxide induced a significant reduction in cell viability, and this reduction was effectively prevented by treatment with C-PC in the low micromolar concentration range. C-PC displayed a strong inhibitory effect against an electrochemically generated Fenton reaction. It was concluded that C-PC is a potential neuroprotective agent against ischemic stroke, resulting in reduced neuronal oxidative injury and protection of mitochondria from impairment [105]. It seems that the neuroprotective effect of PC is related to its antioxidant properties. However, the anti-inflammatory and immuno-modulatory properties could also contribute to its neuroprotective properties [104]. 

It was reported that orally administered C-PE exhibited favorable effect on hepatocellular, hepatobiliary, kidney and redox biomarkers against CCl_4_-induced toxicity in rats [106]. It was concluded that orally administered C-PE could be broken down in the gastrointestinal tract by proteolytic enzymes into low-molecular-weight proteins and bilirubin, and thus mediate the pharmacological effects. Ou et al. [107] reported that C-PC was effective in vitro and in vivo in protecting against CCl_4_-induced hepatocyte damage. A possible mechanism is that C-PCs protect the hepatocytes from free radicals’ damage induced by CCl_4_. C-PC may be able to block inflammatory infiltration through its anti-inflammatory activities by inhibiting TGF-β1 and HGF expression in in CCl_4_-induced hepatic damage.

### 4.6. Immunomodulatory Effects

It has been accepted that the ability of immune regulation is key for the body against various diseases. The effects of PBPs against diseases could be attributed to their immunomodulatory properties. An early study found that the survival rate of the tumor-bearing mice which were dietary-supplemented with PC was significantly higher than that of untreated groups. This was consistent with the changes in lymphocyte activity in each group, indicating that PC had certain stimulating and promoting effects on the immune system [108]. Nemoto-Kawamura, C. [109] suggested that PC enhances biological defense activity against infectious diseases through sustaining functions of the mucosal immune system and reduces allergic inflammation by the suppression of antigen-specific IgE antibody. Pentón-Rol et al. [104] demonstrated that C-PC was able to prevent or downgrade experimental autoimmune encephalitis expression and induced a regulatory T cell response in peripheral blood mononuclear cells from multiple sclerosis patients.

The immunomodulatory activity of the PBP may be associated with their antioxidant properties. Ivanova et al. [110] found that PC could stimulated the lymphocyte antioxidant defense system of occupationally exposed subjects. Lee et al. [111] illustrated that PBP may protect cells from oxidative damage by regulating the body’s immunity and increasing its ability to repair cell damage. However, many studies in recent years showed that the immune mechanism of PBP was related to its anti-inflammatory activity at the cellular and even the genetic level. Chen et al. [112] showed that C-PC had the capability to induce secretion of inflammatory cytokines, including TNF-α, IL-1β, and IL-6. Treatment with C-PC also increased proIL-1β and COX-2 protein expression in a dose-dependent manner and rapidly stimulated the phosphorylation of inflammatory-related signaling molecules, including ERK, JNK, p38 and IκB. Grover et al. [113] presented that C-PC exhibited immunomodulatory activities by suppressing the synthesis of pro-inflammatory cytokines, interferon-γ (IFN-γ), and tumor necrosis factor- α (TNF-α) in a dose-dependent manner in Balb/c mice.

Exposure of PC to human mononuclear cells leads to the generation of Treg cells. Such an effect is similar to what is mediated by HO1 induction. BV, the product of HO1, is structurally homologous to phycobilins. In animal cells, BV is rapidly reduced to bilirubin by biliverdin reductase. Interestingly, injection with bilirubin in mice induced Treg cell formation. It was thus proposed that phycobilins would mimic the effects of biliverdin on Treg induction [114].

### 4.7. Photodynamic Therapy

Photodynamic therapy (PDT) is a therapeutic option for various types of cancers, such as skin tumors, lung tumors, oral tumors and stomach tumors. Effective PDT leads to cancer cell damage and death by inducing ROS-mediated damage, vasculature damage, and immune defense activation. Compared with conventional chemotherapy, PDT selectively kills tumor cells but does not damage normal cells.

PDT can mediate cell death directly. The underlying mechanisms include type I and type II reactions. Type I: triplet photosensitizers directly produce ROS, which aew then transformed into O_2_^−^, OH^−^ or H_2_O_2_ to kill cancer cells. Type II: the triplet photosensitizer reacts with triplet oxygen molecules (^3^O_2_) to produce singlet oxygen (^1^O_2_), and then deoxidizes the substrate [61]. A photosensitizer is the critical element during PDT therapy. An ideal photosensitizer is characterized by: 1. High affinity for tumor cells or selective accumulation in tumor tissues, 2. low dark cytotoxicity, 3. high absorption of light ranging from 600 nm to 800 nm, 4. high quantum yield in ROS production, and 5. high light-dependent cytotoxicity [115,116]. Hematoporphyrin and porphyrin derivatives, which have a tetrapyrrole group, are the commonly used photosensitizers in cancer treatment.

Morcos et al. for the first time presented the potential application of PC as a cytotoxic photosensitizer [117]. Cytotoxicity was evaluated by measuring the viability of mouse myeloma cells in culture after incubation with PC (0.25 mg/mL) and irradiation by 300 J/cm^2^ at 514 nm. After 72 h post-treatment, the cells showed 15% viability compared to 69% and 71% for control cells exposed to laser only or PC only, respectively [117]. In C-PC-treated mice, the weight of immune organs, proliferation of immunocytes, and expression of pro-apoptotic Fas protein were increased, whereas the tumor weight and the expressions of anti-apoptotic proteins (NF-kB and P53) and CD44 mRNA were comparatively decreased. When combined with He–Ne laser irradiation, the effects of C-PC treatment were enhanced [118]. Thus, the anti-tumor activities by C-PC-mediated PDT may be related to the facilitation of apoptosis signal transduction. The in vitro photodynamic effect of C-PC against breast cancer cells was demonstrated to be related to ROS production [119]. In the presence of light, C-PC did not exhibit any visible toxicity. Under illumination at 625 nm, ROS mediated by C-PC killed MDA-MB-231 breast cancer cells in a dose-dependent manner. A recent work described a hybrid material through conjugation of C-PC to biosilica for the PDT of tumor-associated macrophages [120]. The conjugation enhanced the relatively weak photodynamic effect of C-PC, leading to a high photodynamic activity under 620 nm laser irradiation. The enhanced photodynamic activity might be attributed to the enrichment of C-PC-biosilica hybrid on the surfaces of tumor-associated macrophages cell.

R-PE and its subunits were reported to exhibit inhibitory effects on mouse tumor cell S180 and human liver carcinoma cell SMC 7721. Compared with the hexamer, subunit of R-PE seemed to be more effective. An in vivo experiment showed that the survival rate of S180 cells decreased from 90% down to 58% with the increase in R-PE concentration from 10 mg/mL to 100 mg/mL. In the same way, the survival rate of S180 cells decreased from 75% to 44.6% by α subunit, 90.6% to 40.1% by β subunit, and 91% to 31% by γ subunit. The β subunit exhibited not only a better PDT effect but also emitted more fluorescence, which can be used a fluorescent marker for detection of binding sites. In addition, its lower molecular size allows the β subunit to enter the tumor cell more easily [121].

Similar to C-PC, APC covalently binds PCB as the chromophore. Laser photolysis and pulse radiolysis study of APC from *A. platensis* showed that 248 nm laser-flash photolysis led to triplet state and radical cations of APC, which were generated by ionization via a monophotonic process. These findings indicated the potential use of APC as type I and type II photosensitizer [122].

Up to date, no research is available on the evaluation of recombinant PBPs as a photosensitizer. As has been stated in Section 3.2, the preparation of PBP from recombinant E. coli is feasible. Notably, recombinant holo-subunits of PBP have smaller sizes, facilitating their accumulation in the tumor sites. In addition, protein engineering techniques make it possible to fuse PBP to target entities, such as antibodies, for improving affinity to tumor cells. In the future, it is of importance to assess PDT effects of the recombinant PBP systematically.

### 4.8. Other Biological Activities

Other activities including antiviral activity, intestinal flora modulation and wound healing stimulation have been reported. APC inhibited the replication of enterovirus 71 and influenza virus cultured in vitro [123]. Shih and Chueh [124] confirmed that APC extracted from *A. platensis* exhibited anti-enterovirus 71 activity and pointed out that its antiviral mechanism is related to the inhibition of virus proliferation and a reduction in cell apoptosis by reducing the synthesis rate of viral RNA. Using high-throughput 16S rDNA sequencing, Qi et al. [125] examined the responses of gut microbiota in H22-bearing mice to dietary recombinant PE (RPE) supplementation. The results showed that dietary RPE could modulate the gut microbiota of the H22-bearing mice by increasing the abundance of beneficial bacteria and by decreasing that of detrimental bacteria among intestinal bacteria. These findings provide evidence for the mechanism by which bioactive proteins affect intestinal nutrition and disease resistance in animals. Apart from the above-mentioned effects, it was presented that C-PC stimulated wound healing through a urokinase-type plasminogen activator-dependent mechanism, although a detailed molecular mechanism is yet to be elucidated [126].

## 5. Conclusions and Future Perspectives

PBPs represent a large family of light-harvesting biliproteins found in cyanobacteria, cryptomonads and red algae. They have been used as food colorants, nutraceuticals and fluorescent probes in immunofluorescence analysis for many years. Increasing reports have described their various health-promoting features, demonstrating the pharmaceutical potentials of these valuable proteins. It is expected that the pharmaceutical application of these proteins could be achieved in the next decades. To this end, systemic work on PBP absorption, transport, metabolization, molecular target and mechanisms of actions need to be further investigated.

The health-promoting effects of PBPs are mainly tested for C-PC obtained from *Spirulina*. Other types of PBPs (APC and PE) and PBPs derived from other algal species such as cyanobacterial *Anabaena marina*, *Aphanizomenon flos-aquae*, *Oscillatoria tenuis*, red algae *Neopyropia yezoensis* (formerly *Porphyra yezoensis*), *Porphyridium purpureum* (formerly *Porphyridium cruentum*) and many other species also exhibit similar bioactivities. These proteins are structurally different from C-PC and may offer novel biological properties contributing their applications. However, production of PBPs from these species on a large scale have not been achieved and the potential applications of these PBPs should be explored intensively.

The production of recombinant PBPs by engineered microorganisms offers an attractive source for PBPs. The processes of microorganism cultivation and PBP purification are easy to perform and scale up, making it possible to prepare a large amount of PBPs at a low cost. The recombinant PBPs, mainly produced as a single subunit either in apo- or in holo-form, are structurally distinct from the native ones. Interestingly, these proteins also exhibit bioactivities such as anti-tumor and anti-oxidant effects. The small size of recombinant PBPs may facilitate the absorption and transportation to the target tissues. In particular, the bioactivities of PBP may be ascribed to its specific domains. It is intriguing to reconstitute and express these domains in heterologous hosts and evaluate their pharmaceutical potentials. However, until recently, such work had been scarcely carried out, possibly due to the obstacles during the preparation of recombinant proteins. A number of recombinant PBPs have been successfully prepared in our lab. We are willing to provide the plasmids and strains contributing to further explorations of these proteins.

## Abbreviations

PBP: phycobiliprotein; APC, allophycocyanin; PC, phycocyanin; PE, phycoerythrin; PEB, phycoerythrobilin; PCB, phycocyanobilin; PUB, phycourobilin; PVB, phycoviolobilin; BV, biliverdin IXα; PcyA, phycocyanobilin: ferredoxin oxidoreductase; PebA, 15,16-dihydrobiliverdin:ferredoxin oxidoreductase; PebB, phycoerythrobilin:ferredoxin oxidoreductase; ROS, reactive oxygen species; TPT, topotecan; COX-2, cyclooxygenase-2; PDT, photodynamic therapy.
